# Ultraviolet Filters: Dissecting Current Facts and Myths

**DOI:** 10.3390/jcm13102986

**Published:** 2024-05-19

**Authors:** Thomas Breakell, Isabel Kowalski, Yannick Foerster, Rafaela Kramer, Michael Erdmann, Carola Berking, Markus V. Heppt

**Affiliations:** 1 Department of Dermatology, Uniklinikum Erlangen, Friedrich-Alexander-Universität Erlangen-Nürnberg, 91054 Erlangen, Germany; thomas.breakell@uk-erlangen.de (T.B.); isabel.kowalski@uk-erlangen.de (I.K.); yannickdorian.foerster@mri.tum.de (Y.F.); rafaela.kramer@uk-erlangen.de (R.K.); michael.erdmann@uk-erlangen.de (M.E.); carola.berking@uk-erlangen.de (C.B.); 2 Comprehensive Cancer Center Erlangen-European Metropolitan Area of Nuremberg (CCC ER-EMN) and CCC Alliance WERA, 91054 Erlangen, Germany; 3 Bavarian Cancer Research Center (BZKF), 91052 Erlangen, Germany; 4 Department of Dermatology and Allergy Biederstein, Technical University (TU) Munich, 80802 Munich, Germany

**Keywords:** UV filters, sunscreen, skin cancer, prevention, octocrylene, benzophenones, marine environment, endocrine disruption, vitamin D, carcinogen

## Abstract

Skin cancer is a global and increasingly prevalent issue, causing significant individual and economic damage. UV filters in sunscreens play a major role in mitigating the risks that solar ultraviolet ra-diation poses to the human organism. While empirically effective, multiple adverse effects of these compounds are discussed in the media and in scientific research. UV filters are blamed for the dis-ruption of endocrine processes and vitamin D synthesis, damaging effects on the environment, induction of acne and neurotoxic and carcinogenic effects. Some of these allegations are based on scientific facts while others are simply arbitrary. This is especially dangerous considering the risks of exposing unprotected skin to the sun. In summary, UV filters approved by the respective governing bodies are safe for human use and their proven skin cancer-preventing properties make them in-dispensable for sensible sun protection habits. Nonetheless, compounds like octocrylene and ben-zophenone-3 that are linked to the harming of marine ecosystems could be omitted from skin care regimens in favor of the myriad of non-toxic UV filters.

## 1. Introduction

The skin, the external barrier of our body, interacts directly with our environment and is constantly exposed to different environmental stressors like ultraviolet (UV) radiation. Solar UV radiation reaching the earth consists of 90 to 95% UVA (wavelengths 320–400 nm) and 5 to 10% UVB radiation (280–320 nm). UVC radiation (280–100 nm) is absorbed by atmospheric ozone, and thus does not add to ambient sunlight. The biological effects of UV radiation on the skin are known: The acute effects include erythema, edema, sunburn and photoimmunosuppression. The chronic effects comprise photoaging and carcinogenesis. Due to its longer wavelengths, UVA radiation reaches into the dermis, inducing indirect photosensitizing reactions and damaging DNA via reactive oxygen species. UVB radiation is predominantly absorbed by the epidermis, yet directly causes molecular rearrangements in the DNA. UV-induced photolesions that interfere with transcription, DNA replication and base pairing then cause characteristic UV signature mutations [1]. In order to reduce the damage UV radiation inflicts on the skin, thorough and consequent photoprotection habits are essential, especially in children, as they are most vulnerable [2,3,4].

Skin cancer is the most common malignancy in Caucasians, with the incidence of non-melanoma skin cancer (NMSC) being 18 to 20 times higher than melanoma [5]. Skin cancers in the United States (US) population are higher in number than all other cancer entities combined [6]. In the US, melanoma incidence has increased by over 320% since 1975. Following the introduction of targeted and immunotherapy agents in the last decade, the overall 5-year survival of melanoma patients has risen to over 90% in the US. Nevertheless, the 5-year survival rate for stage IV (distant metastasized [7]) disease remains under 30% [8].

A 2015 study estimated the cumulative annual direct healthcare costs for melanoma and NMSC in Australia, New Zealand, Denmark, Sweden, the UK, Germany, France, the US, Canada and Brazil to be greater than EUR 1.5 billion [9]. Another study from 2011 states an average annual total cost of USD 8.1 billion for skin cancer [10]. As the incidence of skin cancer is continuously rising, this number can be expected to have increased since then, putting a substantial economic burden on global healthcare systems, especially in countries with higher socio-economic development and older populations regarding NMSC [5,9].

Exposure to solar UV radiation is the most important preventable risk factor leading to skin cancer [6] and 80–90% of skin cancers are linked to UV exposure [11], yet under 40% of US Americans practice adequate UV protection [8].

There are multiple effective photoprotection strategies, including avoiding solar exposure or wearing protective clothing. In the US and Europe, topical application of sunscreen remains the most common method of sun protection and skin cancer prevention [12,13]. In Australia, skin cancer prevention programs like SunSmart recommend a multifaceted approach, including staying indoors for peak UV exposure hours; policies for hat-wearing and shade provision in child-care centers, primary schools and workplaces; the introduction of sunscreen tax deductibility for outdoor workers; the recommendation of long-sleeved sun-protective swimwear; a ban on tanning beds; the provision of UV forecasts in weather reports; and grants for community shade in addition to the availability of more effective sunscreens that extend protection time and offer UV protection with broader coverage [14]. In 2007, the Cancer Council of Australia updated their 1981 health campaign “Slip, Slop, Slap” to “Slip, Slop, Slap, Seek, Slide”. The aim was to playfully motivate children to slip on a shirt, slop on sunscreen, slap on a hat, seek shade and slide on some sunnies [15], thereby adhering to healthy UV protection strategies.

In recent years, public awareness of sun safety and the damaging effects of sunlight has continuously grown, resulting in an increased intentional use of UV filters. The global sunscreen market is predicted to rise from USD 11.6 billion in 2018 to USD 24.4 billion by 2029 [16,17]. The first UV filters were developed in 1928 and their effectiveness and safety were proven by 1956 [17]. To standardize the measurement of sunscreen efficacy, the sun protection factor (SPF) was developed in 1974 [17,18]. SPF is defined as “the ratio of the smallest dose of UVB radiation required to produce minimal erythema on sunscreen-protected skin compared to the necessary dose of UVB to produce the same amount of erythema on non-protected skin” [19]. The sunburn protection of a sunscreen increases with a higher SPF [20].

The American Academy of Dermatology advises using water-resistant sunscreen with broad-spectrum protection, including UVA and UVB coverage, and at least SPF 30 for optimal safety [21]. It is recommended to apply 2 mg of sunscreen per square centimeter of skin. This amount can be achieved either by the teaspoon rule, using one teaspoon to cover the face, or by reapplication within an hour [22].

Sunscreen protects the skin from UV radiation and its adverse effects. Simultaneously, sunscreens also have to fulfill additional standards: safety concerns, skin tolerability, personalization of use and sustainability already play an important role and will only grow more relevant in the future [2,23].

In this review, we discuss the current controversies surrounding UV filters.

## 2. Classification of UV-Protective Filters

There is a plethora of sunscreen products available on the market, divided into two main types of ultraviolet (UV) filters: organic (chemical) and inorganic (physical, mineral) [24].

**Figure 1:**
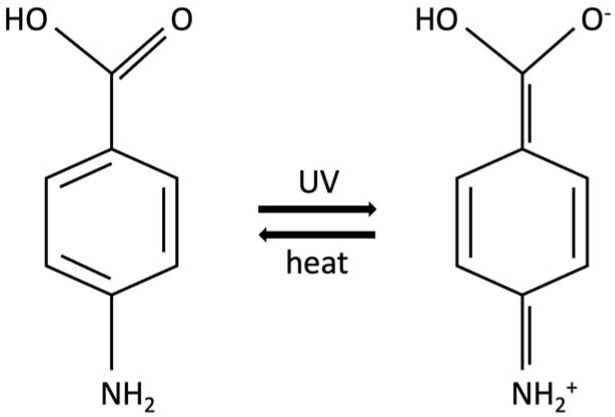
UV radiation leads to the excitation of the conjugated electrons of the organic UV filter 4-aminobenzoic acid (PABA). The polarized molecule spontaneously returns to the ground state by emitting heat or non-damaging wavelengths of light.

Organic UV filters absorb highly energetic UV rays and turn them into non-damaging wavelengths of light or heat, which are then released from the skin. Most organic UV filters are organic compounds containing one or more aromatic rings. In the ground state, their conjugated (delocalized) electrons are at the lowest possible energy levels. After absorbing a distinct amount of energy, electrons may jump into an excited state, leading to the transitory polarization of the molecule. The excited molecule then spontaneously returns to the ground state by emitting heat or long-wavelength radiation [25] (Figure 1). The absorption maximum is molecule-specific and is usually within the spectrum of either UVA or UVB [1,26]. Therefore, commercially available sunscreen products contain a combination of various organic UV filters to provide broad-spectrum UV protection. Sunscreens can also be categorized by their vehicles into lotions, sprays, gels and sticks [18].

Organic UV filters must also meet requirements beyond effective light-absorbing capacity. The excited state is highly energetic, which may cause phototoxic effects due to chemical reactions with adjacent tissue. Therefore, the excited state should be transient, and its chemical structure optimized for sufficient tissue tolerance. Molecules also have to be lipophilic to allow sufficient penetration into the skin, and preferentially 50% of the initial SPF should still be present after contact with water, hence requiring good water resistance. Organic UV filters should be applied at least 15 min before immersion in water to allow sufficient absorption into the skin. Many common organic UV filters that meet the abovementioned criteria are derived from aminobenzoic acids, ethyl cinnamates, salicylic acids and benzophenones [25].

Physical UV filters have a different mechanism for UV protection, namely reflecting and scattering UVA and UVB radiation. They are usually metallic compounds, such as titanium dioxide (TiO_2_) and zinc oxide (ZnO) [18,27]. In contrast to organic UV filters, the molecules comprising physical UV filters are inert, hence not posing the risk of photoallergic or phototoxic side effects and being less irritative for the eyes. The full SPF is available immediately after application. However, because the molecules do not penetrate the skin, water resistance is often inferior compared to organic UV filters. The formula of sunscreen containing physical UV filters is often thicker and the large particle size and high refractive indices of both TiO_2_ and ZnO leave a white, often undesirable residue on the skin. Engineering these compounds into nanoparticles leads to less scattering of visible light and improvement in cosmetic appearance, resulting in improved consumer tolerability. While it was initially suspected that topically applied nano-sized particles could be absorbed systemically, it has been demonstrated that due to their rather large diameter of around 80 nm, their penetration into the skin does not exceed further than the stratum corneum in the epidermis [28,29]. Absorption into the body is thus not to be expected [30].

Modern sunscreen products often contain further active ingredients to prevent UV-induced skin aging, erythema, wrinkling and mutagenesis. This includes classic antioxidants like ascorbic acid and exogenous DNA repair enzymes such as photolyase that restore DNA integrity after topical application [31].

## 3. Regulatory Issues

Regulations of sunscreen products differ from one country to another. While the US Food and Drug Administration (FDA) regulates sunscreens as over-the-counter (OTC) drugs that are subject to FDA regulations [32], the UV filters that are allowed in the European Union (EU) are listed in Annex VI to the EU Cosmetics Regulation [33] that label sunscreen products as cosmetics. In Australia, sunscreens are classified as therapeutic goods by the Therapeutic Goods Administration (TGA) under the Therapeutic Goods Act 1989 [34]. According to the FDA monograph, there are 16 UV filters approved in the US compared to 29 agents in Australia and 30 in the EU [33,34,35].

The FDA further classifies the existing UV filters into three categories: TiO_2_ and ZnO are classified as category I, which is generally recognized as safe and effective (GRASE). Category II includes 4-aminobenzoic acid (PABA) and trolamine salicylate and is not GRASE. For category III, there are inconclusive data to determine GRASE status and the FDA is currently researching more data on the remaining substances, thus not considering them as unsafe. Due to this strict regulation, no new UV filters have been approved in the US since 1998 [32,36]. Of the sixteen approved, only eight (oxybenzone (benzophenone-3, BP-3), avobenzone, octinoxate (OMC), octisalate, homosalate, octocrylene, TiO_2_ and ZnO) are regularly used and only avobenzone and ZnO offer UVA protection [37].

The sunscreen category descriptions based on SPF also differ from one country to another. In Australia, SPF levels from 4 to 14 are considered to offer low protection, 15 to 29 medium protection, 30 to 59 high protection and 60 or higher very high protection. According to the EU regulations, SPF 6–14 offers low protection, 15–29 medium protection, 30–49 high protection and 50 or more ultra-high protection. In contrast, the FDA does not classify SPF levels into categories, merely recommending products with an SPF of at least 15 and 30 to 50 in fair-skinned individuals [38].

Regarding UVA protection in particular, the Japan Cosmetic Industry Association (JCIA) introduced a protection grade of UVA (PA) classification, ranging from PA+ (low) to PA++++ (high). This classification is based on an in vivo persistent pigment darkening method [39,40].

An overview of the organic and physical UV filters that are approved in the EU, Australia and the US as of March 2024 is displayed in Table 1.

## 4. Is Sunscreen an Endocrine Disruptor?

Various organic UV filters are absorbed systemically and may therefore affect endocrine processes, hence being classified as endocrine active chemicals (EACs) [49]. 

Some of the more relevant UV filters are benzophenones or derived from cinnamates or camphor.

A Danish study by Janjua et al. on 32 healthy volunteers showed systemic absorption of a representative of each of these categories (BP-3, OMC and 4-methylbenzylidene camphor (4-MBC)) [50]. Similarly, in a US randomized clinical trial on 24 patients run by Matta (affiliated with the FDA) et al., all sunscreens applied during the study (avobenzone, BP-3, octocrylene and ecamsule) were identified in blood plasma [51]. Using a similar protocol, the same group detected avobenzone, BP-3, octocrylene, homosalate, octisalate and octinoxate in the blood serum of the 48 randomized patients in another study. They also performed skin strippings that revealed the persistence of UV filters in the skin at 21 days after initial exposure [52,53]. Another study by the same group in the same setup produced similar results and additionally identified the three substances in urine [54]. At least one of the nine organic UV filters tested (BP, BP-1, BP-2, BP-3, BP-7, 4-HBP, 4-MBP, 4-MBC, 3-BC) was present in the seminal fluid of 45% of 300 healthy Danish men between the ages of 18 and 29, only 6 of whom had applied sunscreen within 48 h prior to testing [55].

In a study of 200 pregnant women, benzophenones (BP-1, BP-3, 4-MBP, 4-HBP) were detected in human amniotic fluid, urine and fetal and cord blood cells [56]. A Spanish study showed an accumulation of BP-4 in post-partum placenta samples [57]. In a Swiss study, the UV filters (OMC, octocrylene, 4-MBC, homosalate, BP-2, BP-3, OD-PABA, 3-BC) were detected in 46 out of 54 or 85.19% of breast milk samples, with the rank order of frequency of detection corresponding to that of reported use of these filters [58].

These exemplary findings demonstrate the systemic absorption of UV filters in various compartments of the human body, a prerequisite for the potential endocrine effects discussed below.

### 4.1. Gonadocorticoid Effects

Benzophenones display estrogenic and antiandrogenic activity: In an in vitro study analyzing 18 UV filters and one metabolite (including 4-MBC, BP3, BP4, OMC, octocrylene, homosalate, PABA, OD-PABA and PEG25-PABA), all compounds showed hormonal activity, most of them on multiple targets. Agonistic action was shown on both the human estrogen receptor alpha (hERα) and the human androgen receptor (hAR). Anti-estrogenic and antiandrogenic activities were also observed, with UV filters reducing estradiol activity in the hERα assay and inhibiting 4,5-dihydrotestosterone (the most potent androgen [59]) activity [60]. BP-3, homosalate, 4-MBC, OMC and OD-PABA showed estrogenicity in vitro, increasing cell proliferation in MCF-7 breast cancer cells [61]. BP-3 and 4-MBC also increased uterine weight in a uterotrophic assay using immature Long–Evans rats [61]. In a murine in vivo study comparing OMC, 4-MBC and the endogenous hormone estradiol-17β (E2) to ovariectomized controls, all three affected fat and lipid homeostasis, reducing weight gain and the size of fat depots. OMC and 4-MBC additionally reduced serum triglycerides, while E2 and OMC reduced serum cholesterol and low- and high-density lipoproteins. 4-MBC additionally inhibited serum T4, resulting in increased serum TSH levels: findings that indicate more complex effector mechanisms than simply estrogen-receptive effects [62]. (See Section 4.3).

Estrogenic effects were confirmed in various studies [63,64,65,66,67,68] and are additionally relevant in evaluating potential carcinogenic effects, which are discussed below.

A cross-sectional study by Scinicariello et al. and a prospective cohort study by Aker et al. demonstrated an association between BP-3 and testosterone as well as sex-hormone-binding globulin (SHBG [69]) levels [70,71,72].

### 4.2. Developmental Toxicity

In a Danish in vitro study, the UV filters 4-MBC, 3-BC, meradimate, amiloxate, octisalate, Benzylidene Camphor Sulfonic Acid, homosalate, OD-PABA, BP-3, OMC, octocrylene, avobenzone and Diethylamino Hydroxybenzoyl Hexyl Benzoate showed progesterone-mimicking effects by influencing calcium signals. This resulted in an increase in sperm penetration into a viscous medium and the induction of the acrosome reaction (a calcium-dependent prerequisite for spermatozoa to successfully fertilize the oocyte [73]) [74,75]. 3-BC and 4-MBC led to a delay in male puberty and a reduction in prostate weight in Long–Evans rats [76]. Female sexual behavior was also influenced by these two UV filters, reducing proceptive as well as receptive behavior and increasing rejection behavior towards males [77]. BP-2 inhibited spermatocyte and oocyte production and development in mature fathead minnow fish (Pimephales promelas) [78]. It has been reported that 4-MBC delayed male puberty, decreased adult prostate weight and slightly increased testis weight in rats after both the parent generation and offspring were exposed [67].

Whilst these data are derived from in vitro and animal in vivo studies, as mentioned above, Janjua et al. carried out a human single-blinded study with 15 young males and 17 postmenopausal women. After a week of applying a basic cream formulation lacking active ingredients (2 mg/cm^2^) to the whole-body surface once a day, participants applied a UV filter mix containing 10% each of BP-3, OMC and 4-MBC following the same protocol. All three compounds were detected in blood, plasma and urine. The observed minor significant differences in testosterone, estrogen and inhibin B levels between the two weeks were interpreted as chance findings due to mass significance as well as normal biological variations rather than effects caused by the tested UV filters. Janjua at al. hence inferred that in their study the absorbed UV filters had not been capable of disturbing the homeostasis of endogenous reproductive hormones [50].

Four case–control and prospective cohort studies by Buck et al., Chen M et al. and Chen X et al. showed no association between BP-3 and fertility in humans [72,79,80,81,82].

### 4.3. Thyroid Interference 

As estrogen receptors are expressed along the hypothalamic–pituitary–thyroid axis [49], the effects of UV filters on thyroid function have also been studied. The results revealed more complex effects than merely estrogenic ones on thyroid function:

Schmutzler et al. determined that BP-3 and OMC displayed thyroid-receptor-mediated transcriptional activation in vitro; 4-MBC decreased iodine uptake, increased TSH and decreased thyroxine (T4, the prohormone to the active form triiodo-thyronine (T3) [83]) in adult ovariectomized rats [84]. Klammer et al. reported similar results for OMC which additionally decreased triiodo-thyronine (T3) levels and expression of the TSH receptor [49]. A study by Maerkel et al. showed 4-MBC increased both the relative and absolute weight of thyroids in adult mice when both they and their parent generation were treated with 4-MBC [67]. Axelstad et al. exposed the parent generation of rats to OMC, which resulted in reduced T4 levels in the dams and their male offspring. The group could not, however, corroborate the expected effects of hypothyroxinemia on auditory function, learning abilities and motor activity from a previous study [85,86]. In a study by Cahova et al., rainbow trout (Oncorhynchus mykiss) showed an increase in T4 and the downregulation of the genes responsible for thyroid hormone regulation when exposed to OMC [87]. 

Regarding human data, Janjua et al. evaluated the effect of BP-3, OMC and 4-MBC following the same protocol as mentioned in the Section 4.2. The observed minor significant differences in T4, T3 and thyroxine-binding globulin levels were interpreted in the same way as above and the group concluded that in their study UV filters had not been capable of disturbing the homeostasis of human thyroid hormones, in contrast to the data derived from the animal models [88].

Four further studies (case–control, cross-sectional, prospective cohort and retrospective cohort) by Aker et al., Przybyla et al. and Kim et al. found either no significant or contradictory data on the association between BP-3 and thyroid hormone levels in humans [70,72,89,90,91].

UV filters are absorbed systemically through the environment or topical application. They have been proven to affect hormonal pathways in vivo and in vitro in various animal species. Still, the few studies evaluating the hormonal effects of UV filters in the human body could neither prove nor falsify whether they affect the endocrine system. Hence, the data on the endocrine effects of UV filters in humans are currently inconclusive.

## 5. Does Sunscreen Harm Marine Ecosystems and the Environment?

UV filters are present in several environmental compartments, including surface water, groundwater, wastewater, sediments and biota [92], and can expose the organisms inhabiting them to potentially disruptive or damaging effects.

Due to their extensive use in cosmetics, plastics, paints, textiles and many other industrial products, organic UV filters occur ubiquitously in fresh and marine aquatic systems [93]. They enter aquatic environments through wash-off during showering, through the laundering of garments via wastewater treatment plants (indirect input) and through swimming and bathing in bodies of water (direct input) [94]. It is estimated that more than a quarter of applied sunscreen washes off during activities in the water [95]. Additionally, oral drugs and their metabolites are excreted via urine, feces or sweat before entering aquatic systems, leading to possible uptake in marine organisms in coastal waters [96].

Organic UV filters have been detected in numerous water systems, including marine sediments, all around the globe: the Okinawa islands (Japan), Chesapeake Bay (the largest estuary in the United States [97]), Lac Bay (Southern Caribbean), the Canary Islands (Spain), Oahu, Hawaii (USA), the Pearl River Estuary (China), the Baltic Sea (Germany), Erebus Bay (Antarctic), the Virgin Islands (USA), Hong Kong, South Carolina (USA), Liguria (Italy), the Mediterranean Sea (Spain), the Yellow Sea (China), Oslofjord (Norway), Almuñecar (Mediterranean Sea, Spain), the Adriatic Sea (Italy), West Coast (Colombia), West Coast (Chile), Tokyo Bay (Japan), Cadiz Bay (Spain), Huelva Estuary (Spain), Eastern Mediterranean Coast (Lebanon) [98], Kenting National Park (Taiwan) [99], Korean seawater [100] and soil [101], Japanese rivers and lakes [102], West Indies nearshore waters [103], Australian wastewater effluent [104] and influent [105], both fresh- and marine waters in Norway [106], and water treatment plants across Brazil [107], to name only a fraction of the abundance of environmental systems that organic UV filters have been identified in. The presence of UV filters in the Arctic suggests dissemination via ocean currents [108].

In a Swiss study, OMC was detectable in all samples of macroinvertebrates, fish and cormorants taken from the River Glatt and Lake Greifen, with similar levels at all trophic levels indicating a trend for bio-/trophic magnification in the food chain. Both this phenomenon and the prerequisite for it, bioaccumulation, can be explained by the hydrophobic/lipophilic nature of most UV filters [93,98,109,110]. UV filters can thus transfer from aquatic to non-aquatic ecosystems, through terrestrial predators like birds or bears feeding on aquatic animals (trophic transfer). Organic UV filters have been detected in the tissues of Eurasian sparrowhawks, French owls and Greenlandic eagles [111]. In a Spanish study retrieving the unhatched eggs of seven different bird species, including the white stork (Ciconia ciconia) and western marsh harrier (Circus aeruginosus), UV filters were present in the samples of all birds [112].

There are substantially less data on the occurrence and distribution of physical UV filters. Nanoparticles are likely to have a widespread geographic distribution, yet considering the dearth of available information regarding the ecosystem-based fate of these compounds and the various sources of nanoparticles besides UV filters, detailed data of occurrence are hardly obtainable. Nanoparticles enter the environment through industry, emissions, deposition, bioremediation and agriculture, in particular through wastewater. Concentrations of TiO_2_ nanoparticles (nTiO_2_), for example, can merely be estimated through simulations. In situ experimental results are often vague and inconclusive due to the complexity and unpredictability of natural systems. Research is thus conducted using miniature ecosystems called micro- and mesocosm systems [113].

There are little data regarding the persistence and degradation of UV filters in marine ecosystems. UV filters are subject to transformation via photodegradation through sunlight exposure and biodegradation by microbial communities [98]. When UV filters are applied prior to immersion into chlorinated water (i.e., swimming pools), they are exposed to potential interactions with materials of human origin like urine, sweat, cosmetics, skin cells and hair. This can result in the creation of disinfection by-products (DBPs) [114] that occur when disinfectants react with organic and inorganic matter in the pool [115]. Chlorine is a potent oxidizer and the resulting DBPs are more toxic than the corresponding parent UV filters. For instance, when BP-3, dioxybenzone and sulisobenzone reacted with chlorinating agents, their UV absorbance was reduced and the former two additionally induced cell death in vitro [116].

### 5.1. Coral Reefs

It is estimated that around 4000 to 14,000 tons of UV filters are released in reef areas every year [95,108].

Coral reefs are hotspots of marine biodiversity and directly sustain half a million humans. More than half of them are threatened by natural and anthropogenic impacts. They are vulnerable to environmental changes like fluctuations in water salinity levels, temperature and pH [117]. One of the results of this is coral bleaching, i.e., the loss of symbiotic zooxanthellae hosted within scleractinian corals. Organic UV filters have been demonstrated to induce the lytic viral cycle in symbiotic zooxanthellae with latent infections, resulting in complete and rapid bleaching of hard corals in the Atlantic, Indian and Pacific Oceans and the Red Sea, even at low concentrations [95]. A similar effect has also been observed in bacterioplankton when testing a commercially available sunscreen with a filter system comprising both physical and organic UV filters (e.g., octocrylene, ethylhexyl salicylate and titanium dioxide) [118].

To preserve marine ecosystems, the US state of Hawai’i banned the use of BP-3 and OMC and also discussed the banning of avobenzone, homosalate, octisalate and octocrylene. The latter reduces the ability of coral symbionts to photosynthesize and can be toxic to a variety of aquatic organisms, including corals, fish, mammals and plants. Avobenzone also disrupts photosynthesis and fat metabolism, which is essential for corals to survive [119,120]. Other regions followed suit: Aruba, Palau, Bonaire, the US Virgin Islands and also Key West, Florida banned selections of BP-3, OMC, octocrylene and 4-MBC while physical UV filters remained unmentioned, despite their known toxicity to aquatic life [121].

The exposure of corals (*Acropora* spp.) to ZnO induced severe and fast coral bleaching due to the alteration of the symbiosis between coral and zooxanthellae, while two modified forms of TiO_2_ (Eusolex^®^T2000 (Merck KGaA, Darmstadt, Germany) and Optisol™ (Oxonica Ltd. and UK Nanotechnology Company, Aylesbury, UK)) barely had any detrimental effects on corals, implying better eco-compatibility [122]. ZnO nanoparticles (nZnO) showed toxicity in macroinvertebrates: the isopod Cymodoce truncate, the amphipod Gammarus aequicauda and the sea urchin Paracentrotus lividus [123]; two marine crustaceans: copepod Tigriopus fulvus and the amphypod Corophium insidiosum [124]; and the crustacean Daphnia magna and zebrafish (Danio rerio) [125]. Similar effects have been observed with nTiO_2_ inducing oxidative damage and genotoxic effects in the marine mussel Mytilus trossulus [126].

### 5.2. Other Marine and Terrestial Animals

4-MBC induced malformations, decreased heart rate and altered neurotransmission during the embryonic development of zebrafish [109]. Octocrylene disturbed histologic development and showed hormonal activity in this organism [127], as well as influencing the transcription of genes related to developmental and metabolic processes in the liver [128]. BP-4 interfered with the expression of genes involved in hormonal pathways and steroidogenesis in zebrafish [129]. In harlequin flies (*Chironomus riparius*), a reference organism in aquatic toxicology, 4-MBC and BP-3 affected key hormonal receptors and regulatory genes [93,130]. BP-3 crossed the blood–brain barrier following dermal application in rats, raising oxidative stress, inducing apoptosis in the brain and altering glutamate signaling [131]. Homosalate, avobenzone and octocrylene killed 54%, 64% and 88%, respectively, of exposed brine shrimp (*Artemia salina*) at a concentration of 2 mg/L [132]. Avobenzone and octocrylene induced behavioral impairment like a decreased phototactic response, changes in metabolic rate and subsequently death in water fleas (*Daphnia magna*), a freshwater invertebrate [133]. A Brazilian study showed an accumulation of octocrylene in the livers of 56 Franciscana dolphins (*Pontoporia blainvillei*), suggesting biomagnification [134].

Under the influence of UV radiation, nanoparticles can produce radicals that endanger aquatic organisms [135]:

When exposed to nTiO_2_, crayfish (*Procambarus clarkia*), an in vivo model often used as a bioindicator of water pollution, showed alteration in antioxidant activities and severe histopathological changes [136]. Long-term exposure of zebrafish to nTiO_2_ caused oxidative damage and subsequently upregulated antioxidant enzymes [137]. nTiO_2_ was also toxic to mussels (*Mytilus coruscus*), which are suspension feeders and hence a unique target group for nanoparticle toxicity [138].

nZnO showed toxicity in two marine diatoms (*Skeletonema costatum* and *Thalassiosia pseudonana*), two crustaceans (*Tigriopus japonicus* and *Elasmopus rapax*) and the medaka fish Oryzias melastigma, which is hypothesized to be attributed to dissolved Zn^++^ ions [139]. nZnO also induced DNA damage in the spermatozoa of sea urchins (*Paracentrotus lividus*) [140].

Initiatives like Save the Reef aim to sensitize consumers to omit the controversial UV filters BP-3, OMC, octocrylene, homosalate, 4-MBC, PABA, nTiO_2_ and nZnO while advocating for the use of physical non-nano UV filters like TiO_2_ and ZnO [141]. Cosmetics and sunscreen manufacturers have reacted to the abovementioned findings, banning UV filters like BP-3, OMC and octocrylene from their formulations. They are increasingly introducing slogans like skin protect/ocean respect, which come along with promises of non-toxic, non-water-soluble UV filters and environmentally friendly ingredients. They also test their filter systems on algae, coral and shrimp or fund studies with partners of external organizations, concluding that their products have been proven to have no adverse effects on marine life. Terms used in marketing include Committed to Respecting Marine Life, reef-safe and reef-friendly [132,142,143,144,145,146,147]. Miller et al. criticized these bans and the terms reef-safe and reef-friendly in the absence of standardized testing schemes for scleractinian corals [121].

In summary, UV filters are present in water systems all around the globe; they are proven to induce damage to corals and can be toxic to the organisms inhabiting marine and freshwater ecosystems. Similarly to pharmaceuticals entering aquatic systems, concentrations of UV filters can be expected to be strongly diluted by seawater [96]. The influx of sunscreen that enters global waters through both direct and indirect input is not avoidable, nor is an effort to generally reduce sunscreen use a sensible consequence. A feasible option might be to advocate for the omission of certain proven toxic compounds from sunscreen products, a notion cosmetics manufacturers have already gotten on board with, while simultaneously focusing on the development of biodegradable UV filters [117]. 

## 6. Is Sunscreen Neurotoxic?

Organic UV filters with reported neurotoxicity include OMC, BP-3, BP-4, 4-MBC and octocrylene. In 2017, Ruszkiewicz et al. summarized the neurotoxic effects of organic and inorganic UV filters known at that point in time [148].

The safety of benzophenones (BPs) is widely discussed due to their proven permeability through the skin, the placental barrier and even the blood–brain barrier [131,149,150]. These findings lead to questioning the effects of BPs on the developing nervous system. Fediuk et al. presented signs of neurotoxicity of BP-3 in cultures of rat neurocortex cells [151]. Wnuk et al. conducted an in vivo study in which mouse embryos were exposed to environmentally relevant doses of BP-3 prenatally that showed permeability through the blood–brain barrier, alternations in autophagy and dysregulations in neurogenesis- and neurotransmitter-related genes [150]. The same group also showed that BP-3 inflicted neurotoxicity and activated apoptosis via an intrinsic pathway in vitro [152]. Huo et al. reported increased concentrations of BP-3 in urine samples from mothers of children with Hirschsprung’s disease, an enteric neuropathy [153]. This finding is supported by the previous study by Wang et al., in which BP-3 was associated with abnormal development of the enteric nervous system in zebrafish embryos [154]. Li et al. showed that 4-MBC can lead to neuronal defects through association with neuronal development in zebrafish embryos [155]. Broniowska et al. presented a study on SH-SY5Y neuroblastoma cells in which both BP-3 and 4-MBC adversely affected the viability of nerve cells [156]. Chu et al. showed that OMC can be neurotoxic in adult male and embryo-larval zebrafish, which is assumed to be associated with induced hypothyroidism [157]. In accordance with the study by Nataraj et al., OMC blocks acetylcholinesterase (a neurotoxicity marker [158]) in zebrafish embryos [159].

The inorganic UV filters TiO_2_ and ZnO have also shown negative effects on the nervous system: Jin et al. showed a potential risk for the development of Parkinson’s disease after exposure to high doses of ZnO in zebrafish larvae and human neuroblastoma cells [160]. Yet, the safety statement of the European Commission (SCCS) states that the amount of resorbed ZnO from the topical application of sunscreen products is insignificant and therefore not a risk for the consumer [161].

Interestingly, Ruszkiewicz et al. pointed out that there are signs of neurotoxicity for the widely used organic filter TiO_2_. Multiple reports showed an accumulation of TiO_2_ in the brains of mice or rats [162,163,164,165] and in zebrafish embryos [166]. In an in vitro study using embryonic rat brains, TiO_2_ NPs decreased neuroblasts and increased gliosis. When rats were injected intraperitoneally, TiO_2_ crossed the blood–brain barrier and induced oxidative stress, cellular lysis, neuronal apoptosis and inflammation in vivo [167].

UV filters show neurotoxicity in vitro and exert damage to the nervous system in vivo in animals. The accumulation of these chemicals in ecosystems exposes organisms (including humans) to their potentially detrimental effects. A definitive statement on UV filters’ potential neurotoxicity in humans, therefore, cannot be made given the current state of research.

## 7. Is Sunscreen Carcinogenic?

The data on UV filters as carcinogens are scarce.

Within organic UV filters, benzophenone is listed as a possibly carcinogenic substance and is therefore prohibited as a flavoring substance by the FDA [168]. The decision to ban benzophenone from oral usage was based on the finding that high concentrations in the food of exposed mice and rats led to an increased incidence of renal tumors and possibly leukemia and sarcoma [169]. In 2021, Down et al. and Foubert et al. stated that octocrylene metabolizes to benzophenone over time, which indicates that sunscreen containing octocrylene should not be used after a certain amount of time to avoid any potential carcinogenicity. It should be noted that this study was performed within six weeks and that the aging process was imitated by an accelerated aging protocol, in which the purchased products were stored in an incubator at 40 °C with 75% relative humidity [170,171]. Phiboonchaiyanan et al. showed that BP-3 increased metastasis potential in lung cancer cells [172]. Alamer et al. determined that BP-3, OMC, 4-MBC or homosalate increased the migratory and invasive properties of two human breast cancer cell lines in vitro [173]. Evaluating the available data, the SCCS proclaimed that the use of these UV filters in cosmetic products is safe up to the maximum concentrations shown in Table 1. Rachón et al. discussed an effect on immunity of BP-2 and OMC after showing a Th1/Th2-imbalance with a Th2 shift in vitro [174]. Considering that cytokine dysregulation could be associated with carcinogenesis, this could be a hypothesis for further research [175].

Regarding physical UV filters, a study published in 1985 reported lung tumors in rats induced by prolonged inhalation of TiO_2_ at concentrations overloading lung particle clearance. Based on this finding, the International Agency for Research on Cancer classified TiO_2_ as a potential carcinogen. However, neither other animal species nor humans showed an increased incidence of cancer following TiO_2_ exposure, so the Edinburgh Expert Panel concluded that it posed no cancer hazard to humans, which is congruent with the summary of the original study from 1985 [176,177]. Still, as a precautionary measure, the SCCS recommends that the nanoparticles nTiO_2_ and nZnO should not be used in spray sunscreen that could result in inhalation [161].

## 8. Does Sunscreen Cause Acne?

Acne vulgaris is an inflammatory skin disease of the pilosebaceous follicles. It is the most common dermatosis in the world, with a significantly higher prevalence among adolescents in Western industrialized countries [178]. Patients with acne vulgaris suffer from inflammatory and non-inflammatory lesions reaching from comedones, papules and pustules up to nodules and cysts on body parts with a large number of sebaceous glands like the face, chest, trunk, back and upper arms [179].

The multifactorial pathogenesis of acne includes increased sebaceous gland activity with an excess of sebum production and retention leading to hyperkeratinization of pilosebaceous follicles, hypercolonization of sebum with Propionibacterium acnes and the release of inflammatory mediators [178,179,180]. This results in extremely oily skin and a tendency for blocked pores [181], while still lacking healthy and protective fats on the epidermal surface [182].

There are known endogenous causes for acne, including genetic predisposition, hormonal fluctuation and dysfunction—especially elevated androgen levels—as well as various exogenous causes and aggravation factors, including the use of topical comedogenic substances [183,184], wearing of occlusive clothes, consumption of food with high glycemic index, exposure to stress or excessive solar radiation [179]. Higher temperature and humidity can also induce acne aggravation and flare-ups [184] by causing acute obstruction due to swollen epidermal keratinocytes [185].

It is known that sunscreens can provoke irritation of the skin in a subset of people and can even be comedogenic and acnegenic [186]. Mills and colleagues tested twenty-nine sunscreen formulations by applying them to the external ear canal of albino rabbits. Fourteen, including homosalate and BP-3, were found to be comedogenic. Simultaneous UV radiation augmented their comedogenicity. Interestingly, the vehicles were identified as the cause, while UV filters were demonstrated to be non-comedogenic. Acne provoked by sunscreen can therefore be seen as a subtype of acne cosmetica [183,187].

Over 40 years ago, in 1978, Mills and colleagues assumed that sunbathing worsens acne by increasing the comedogenicity of sebum [188]. Now, sunlight exposure is a proven progression factor in acne vulgaris. Solar radiation and UVB in particular have been shown to promote bacterial proliferation while also inhibiting immune response, therefore driving inflammation, the proliferation of keratinocytes and excess sebum production [180,184]. Additionally, UVA radiation can lead to a thickening of the stratum corneum, modifying the skin microbiome up to dysbacteriosis and causing post-inflammatory hyperpigmentation on acne skin [22].

These phenomena can be prevented by the use of sunscreen. Applying sunscreen not only reduces the number of inflammatory and non-inflammatory acne lesions [180] but also prevents permanent hyperpigmentation from skin irritation and inflammation, especially in individuals with darker skin. Sunscreen additionally protects the skin against photosensitivity and phototoxicity, which can be typical side effects of topical and systemic acne medications [22,186], emphasizing the great importance of UV protection in modern acne therapy.

Modern-day acne therapy not only consists of specific drugs, but includes skin products like cleansers, moisturizers and sunscreen [180]. Photoprotection with non-comedogenic sunscreen is one of the four fundamental parts of patients’ skincare routines, which, in addition to topical medication, helps maintain the structural and functional integrity of the epidermal skin barrier [189], enabling patients to benefit from treatment and decreasing the chances of exacerbation [181]. Photoprotection therefore plays an essential part in assuring a balanced skin barrier and long-term skin health [182].

Patient education also plays an essential role in acne therapy. Regarding the use of sunscreen, people with acne vulgaris or acne-prone skin should be recommended to use light, oil-free, non-occlusive and therefore non-comedogenic sunscreen products like sprays, gels or liquids [186,190]. Oil-based formulations can be aggravating factors and should therefore be avoided in order to maintain a healthy skin appearance [179,181,191].

## 9. Does Sunscreen Cause Vitamin D Deficiency?

Vitamin D deficiency is a rising global health problem [192], affecting a billion people around the globe [193]. Vitamin D plays a crucial role in physiological calcium and phosphate homeostasis and therefore affects human skeletal health. Chronic vitamin D deficiency increases the risk of osteoporosis [193,194] and is furthermore associated with cardiovascular diseases, autoimmune diseases like type 1 diabetes and possibly multiple sclerosis, cancer and depression [193,195,196].

**Figure 2:**
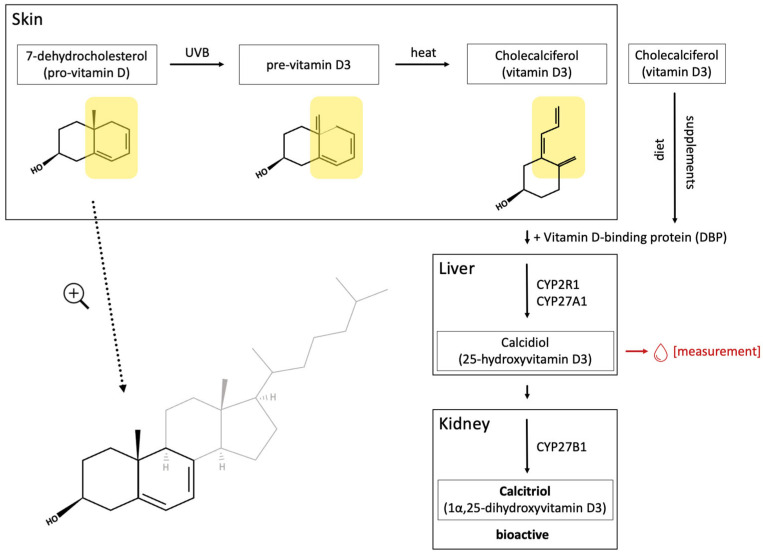
The pathway of vitamin D synthesis. The molecule depicted in the bottom left is 7-de-hydrocholesterol (pro-vitamin D). The relevant positions in the respective steps of synthesis taking place in the skin have been magnified and the exact location of change marked in yellow [210,211,212].

There are two forms of vitamin D: ergocalciferol (vitamin D2) and cholecalciferol (vitamin D3). Vitamin D3 has a higher potency and is the predominant form in the human body [193]. At approximately 90%, the majority of vitamin D production occurs by endogenous synthesis in the skin through sunlight, more precisely UVB radiation exposure [193,197]. UVB photons penetrate the skin and photolyze 7-dehydrocholesterol (7-DHC = pro-vitamin D) to pre-vitamin D3. Secondly, pre-vitamin D3 is isomerized to cholecalciferol (vitamin D3) in a body temperature-dependent reaction [198]. Cholecalciferol, linked to a vitamin D-binding protein (DBP), is released into the bloodstream [199] to be sequentially activated to form calcidiol (25-hydroxycholecalciferol = 25-hydroxyvitamin D3) by the liver and finally converted into the biologically active vitamin D3 (calcitriol = 1,25-dihydroxycholecaliferol) in the kidneys [200]. Kidney or liver diseases may thereby affect vitamin D levels [196] (see Figure 2).

Only small amounts of vitamin D can be ingested, mainly through animal products like dairy products, egg yolk, or fatty fish [193]. As a regular Western diet is often not a sufficient vitamin D source [199], supplementation of vitamin D, especially during winter months, can be beneficial. In the US, vitamin D3 is added to milk to increase oral uptake [18,193,201,202].

Although the ideal serum levels of vitamin D remain controversial, most experts recommend serum levels ≥ 50 nmol/L (≥20 ng/mL) of 25-hydroxycholecalciferol (calcidiol). Lower levels are considered as vitamin D deficiency [196,200,203].

As mentioned above, vitamin D synthesis begins with the help of UVB radiation at a wavelength of 290–320 nm. By blocking UVB rays through sunscreen application, there is a theoretical risk that sunscreen could decrease endogenous vitamin D production, leading to vitamin D deficiency [18,204]. This concern was initially supported by experimental data in vitro and in vivo. Sunscreen blocked cutaneous vitamin D production significantly, and vitamin D levels in the blood were slightly affected [205]. However, real-life situations with long-term sunscreen application (SPF < 50) over several months had no impact on vitamin D status [205]. There are multiple possible reasons for this phenomenon:

Firstly, the average amount of applied sunscreen with 0.8 mg/cm^2^ is significantly lower than the recommended 2 mg/cm^2^. Most of the time, not all sun-exposed body parts are fully covered with sunscreen. Hence, a significant amount of sun can reach the skin before application, or areas with insufficient sunscreen application, and can induce vitamin D synthesis. Normal usage of sunscreen should therefore not result in vitamin D deficiency [197]. It is important to keep in mind that no sunscreen can block 100% of incident UV rays [18] and that photoprotection application may provide a false sense of security in more frequent and prolonged sun exposure [194,205,206]. Apart from sunscreen use, vitamin D synthesis also depends on patient-related factors like skin type or age, sun exposure habits like shade seeking and clothing, and external factors like weather, pollution, or the position and height of the sun due to season, latitude and time of the day. Body surface area (BSA) and time spent outside directly correlate with vitamin D status. Other photoprotection behaviors therefore may have more impact than sunscreen use. The UVB dose (UVB intensity multiplied by exposure time) is the most important factor in vitamin D synthesis [198,200]. Interestingly, the UVB dose necessary for vitamin D3 production in the skin is very small. Regular low-level sun exposure is vastly more effective and beneficial for vitamin D synthesis than excessive UV exposure, additionally reducing the risk of sunburn and skin cancer [207].

Young and colleagues established that sunscreens with a higher UVA protection significantly facilitated vitamin D synthesis, probably by allowing more UVB transmission in comparison to sunscreens with lower UVA protection [208]. This is a convincing argument for wide-spectrum UVA as well as UVB protection [199].

In conclusion, there is no evidence that sunscreen decreases serum vitamin D levels when used in real-life settings [18,204]. However, there are no real-life trials with the currently widely recommended sunscreen products with high sun protection factors (SPF ≥ 50). The impact of long-term sunscreen use with high SPF on vitamin D levels hence remains uninvestigated [204,205]. Regular assessments of serum levels of vitamin D may be recommended if patients are concerned about deficiency. More importantly, sunscreen use reduces the chances of sunburn and prevents skin aging and skin cancer in the long term. Daily use, even on overcast days and in winter, is therefore highly recommended [204,208,209].

## 10. Discussion and Conclusions

UV filters are ubiquitous, and as a result are present in many organisms inhabiting the environment. They can interfere with their development and physiological function and may theoretically affect endocrine processes and the nervous system in humans. These findings therefore necessitate a benefit–risk assessment resulting from the use of UV filters. Sunscreen minimizes the risk of skin cancers, reducing the risks of morbidity and mortality caused by melanoma and NMSC and decreasing the signs of UV-induced photoaging like wrinkles, telangiectasia and pigmentary alterations [11,213].

From a dermatological perspective, the skin cancer risk reduction achieved by the use of sunscreens is the crucial aspect when discussing their use. Until contradictory evidence comes to light, it remains vital to reinforce the clinical recommendations for sunscreen use rooted in biological rationale and clinical evidence [214]. UV filters can have a direct impact on both individual patients and on an epidemiological scale, improving patient outcome and reducing the economic burden caused by skin cancer. These effects are quantifiable and well studied, while the threats UV filters can pose to the human body are not as tangible and remain controversial. The majority of the findings implying their health-related risks are derived from animal in vivo and in vitro studies. While UV filters have also been shown to be absorbed into many parts of the human body, information pertaining to the direct adverse consequences of this accumulation is scarce. Further study is needed to test sunscreens under actual use conditions, regarding factors like differing ratios of body surface area to overall size in infants and children and the application of less sunscreen than recommended. Appropriately designed trials will be necessary to establish a balance of risk and benefit when they are used to prevent skin cancers [214].

Nevertheless, there is increasing evidence of the environmental effects caused by UV filters, which should be seen in a more critical light. Phenomena like coral bleaching may demonstrably and mechanistically be linked to the occurrence of UV filters in aquatic environments. While abandoning the use of sunscreen per se is not a viable option from a skin cancer prevention perspective, the omission of certain UV filters like octocrylene and BP-3 that are proven to have detrimental effects on flora and fauna seems prudent and sensible. The SCCS has reacted to the abovementioned findings, stating that “there is potential risk to human health arising from the use of benzophenone-3 and octocrylene as UV filters in cosmetic products in the concentrations currently allowed” and introducing stricter regulations on the permitted concentrations of these two filters [41,42,43] (see Table 1).

While the public, sunscreen manufacturers and regulators keep a watchful eye on UV filters, it remains to be elucidated whether UV filters actually may cause damage to the human body. Until further research and emphasis on the development of non-toxic and endocrinologically inert UV filters is accomplished, these aspects remain myths rather than facts.

## Figures and Tables

**Table 1:** Approved UV filters in the European Union, Australia and the United States of America [33,34,35,41,42,43,44,45,46,47].

EU	AUS	US	Common/Trade Name	IUPAC Name
–	–	**15 ^N^**	PABA	4-Aminobenzoic Acid
**6**	**6**	–	-/-	Camphor Benzalkonium Methosulfate
**10**	**15**	**15**	Homosalate/Neo Heliopan ^®^ HMS	Homomenthyl Salicylate
**6** ^**†**^	**10**	**6**	Oxybenzone/Neo-Heliopan ^®^ BB	Benzophenone-3 (BP-3)
**8**	**4**	–	Ensulizole	Phenylbenzimidazole Sulfonic Acid
**10**	**10**	–	Ecamsule/Mexoryl ^®^ SX	Terephthalylidene Dicamphor Sulfonic Acid
**5**	**5**	**3**	Avobenzone/Neo Heliopan ^®^ 357	Butyl Methoxydibenzoylmethane
**6**	**6**	**4**	e.g., Mexoryl ^®^ SL	Benzylidene Camphor Sulfonic Acid
**10 ^††^**	**10**	**10**	Octocrylene/Neo Heliopan ^®^ 303	Octocrylene
**6**	–	–	-/-	Polyacrylamidomethyl Benzylidene Camphor
**10**	**10**	**7.5**	Octinoxate	Ethylhexyl Methoxycinnamate * (EHMC/OMC)
**10**	**10**	–	PEG-25 PABA	PEG-25 PABA/Polyoxyethylene ethyl-4-aminobenzoate
**10**	**10**	–	Amiloxate/Neo Heliopan ^®^ E 1000	Isoamyl p-Methoxycinnamate
**5**	**5**	–	e.g., UVINUL T 150 ^®^	Ethylhexyl Triazone
**15**	**10**	–	Silatrizole/e.g., Mexoryl ^®^ XL	Drometrizole Trisiloxane
**10**	–	–	Iscotrizinol/Uvasorb^®^ HEB	Diethylhexyl Butamido Triazone
**4**	**4**	–	Enzacamene/Neo Heliopan ^®^ MBC	4-Methylbenzylidene Camphor (4-MBC)
**5**	**5**	**5**	Octisalate/Neo Heliopan ^®^ OS	Ethylhexyl Salicylate
**8**	**8**	**8**	Padimate O	Ethylhexyl Dimethyl PABA
**5**	**10**	**10**	Sulisobenzone/UVINUL^®^ MS 40	Benzophenone-4, Benzophenone-5 **
**10**	**10**	–	Bisoctrizole/Tinosorb^®^ M	Methylene Bis-Benzotriazolyl Tetramethylbutylphenol (nano)
**10**	**10**	–	Bisdisulizole disodium/Neoheliopan ^®^ AP	Disodium Phenyl Dibenzimidazole Tetrasulfonate
**10**	**10**	–	Bemotrizinol/Tinosorb ^®^ S	Bis-Ethylhexyloxyphenol Methoxyphenyl Triazine
**10**	**10**	–	Parsol ^®^ SLX	Polysilicone-15/Dimethicodiethylbenzalmalonate
**25**	**25**	**25 ^G^**	-/-	Titanium Dioxide (nano) ((n)TiO_2_)
**10**	**10**	–	e.g., Uvinyl ^®^ A Plus	Diethylamino Hydroxybenzoyl Hexyl Benzoate
**10**	**10**	–	e.g., Tinosorb A 2B ^®^	Tris-Biphenyl Triazine (nano)
**25**	**N/A**	**25 ^G^**	-/-	Zinc Oxide (nano) ((n)ZnO)
**5**	–	–	e.g., TriAsorB ^®^	Phenylene Bis-Diphenyltriazine
**3**	–	–	e.g., Mexoryl 400 ^®^	Methoxypropylamino Cyclohexenylidene Ethoxyethylcyanoacetate
**10**	–	–	-/-	Bis-(Diethylaminohydroxybenzoyl Benzoyl) Piperazine (nano)
–	**12**	**12 ^N^**	e.g., Neo Heliopan ^®^ TS	Trolamine Salicylate
–	**5**	**5**	Meradimate/Neo Heliopan ^®^ MA	Menthyl Anthranilate
–	**6**	**3**	Cinoxate/Neo Heliopan ^®^ AV	2-Ethoxyethyl-p-Methoxycinnamate
–	**3**	**3**	Dioxybenzone	Benzophenone-8 (BP-8)

max. approved concentration (%): Ingredient not approved in the respective region; *: 2-Ethylhexyl-4-Methoxycinnamate (EHMC), also known as Octyl-p-Methoxycinnamate (OMC). As discussed by Mitchelmore et al., a homogenous nomenclature, e.g., via the International Nomenclature of Cosmetic Ingredients (INCI), to avoid confusion between names as well as ingredients may be sensible [48]; **: Benzophenone-5 is the sodium salt of Benzophenone-4; **^†^**: 6% in face products, hand products and lip products, excluding propellant and pump spray products; 2.2% in body products, including propellant and pump spray products; 0.5% in cosmetic products other than face products, hand products, lip products, body products, propellant and pump spray products; **^††^**: 9% in propellant spray products; ^G^: generally recognized as safe and effective (GRASE) by the United States Federal Drug Administration (FDA). ^N^: not GRASE; the mentioned trade names are examples chosen at random.

## Data Availability

Not applicable.

## Abbreviations

BP	Benzophenone
DBPs	disinfection by-products
DNA	deoxyribonucleic acid
E2	estradiol
EU	European Union
EUR	Euro
FDA	Food and Drug Administration
GRASE	generally recognized as safe and effective
hAR	human androgen receptor
hERα	human estrogen receptor alpha
JCIA	Japan Cosmetic Industry Association
NMSC	non-melanoma skin cancer
PA	protection grade of UVA
SCCS	safety statement of the European commission
SPF	sun protection factor
TSH	thyroid-stimulating hormone
T3	triiodo-thyronine
T4	thyroxine
US	United States
USA	United States of America
USD	United States Dollar
UV	ultraviolet
UVA	ultraviolet A
UVB	ultraviolet B
UVC	ultraviolet C
